# Insights into the length and breadth of methodologies harnessed to study human telomeres

**DOI:** 10.1186/s40364-024-00668-9

**Published:** 2024-10-22

**Authors:** Tiernan Coulter, Claire Hill, Amy Jayne McKnight

**Affiliations:** Centre for Public Health, Queen’s University Belfast, Institute of Clinical Sciences - Block A, Royal Victoria Hospital, Grosvenor Road, Belfast, BT12 6BJ UK

**Keywords:** Ageing, Association, Bionano, Long-read sequencing technology, Nanopore, PacBio, QPCR, Telomere

## Abstract

Telomeres are protective structures at the end of eukaryotic chromosomes that are strongly implicated in ageing and ill health. They attrition upon every cellular reproductive cycle. Evidence suggests that short telomeres trigger DNA damage responses that lead to cellular senescence. Accurate methods for measuring telomeres are required to fully investigate the roles that shortening telomeres play in the biology of disease and human ageing. The last two decades have brought forth several techniques that are used for measuring telomeres. This editorial highlights strengths and limitations of traditional and emerging techniques, guiding researchers to choose the most appropriate methodology for their research needs. These methods include Quantitative Polymerase Chain Reaction (qPCR), Omega qPCR (Ω-qPCR), Terminal Restriction Fragment analysis (TRF), Single Telomere Absolute-length Rapid (STAR) assays, Single TElomere Length Analysis (STELA), TElomere Shortest Length Assays (TESLA), Telomere Combing Assays (TCA), and Long-Read Telomere Sequencing. Challenges include replicating telomere measurement within and across cohorts, measuring the length of telomeres on individual chromosomes, and standardised reporting for publications. Areas of current and future focus have been highlighted, with recent methodical advancements, such as long-read sequencing, providing significant scope to study telomeres at an individual chromosome level.

## Introduction

Telomeres are repeating noncoding nucleotide sequences of (TTAGGG)_n_ found at the end of eukaryotic chromosomes that preserve DNA structural integrity. Traditional DNA polymerases are incapable of fully replicating the linear genome, causing the “end-replication problem”, where telomeres are truncated throughout a cell’s lifetime [1]. This results in cellular senescence, when cells are metabolically active but do not divide, potentially leading to chromosomal instability [2]. The reverse transcriptase, Telomerase, expressed during development, in highly mitotic and stem-like cells, adds telomeric repeats to offset shortening [2]. Telomerase is reactivated in most cancer cells to allow infinite division [2], with genetic predisposition towards longer telomeres increasing cancer risk [3]. Telomere-induced senescence leads to immortalisation, yet cancer complications in short telomere syndromes are relatively rare [3].

**Fig. 1:**
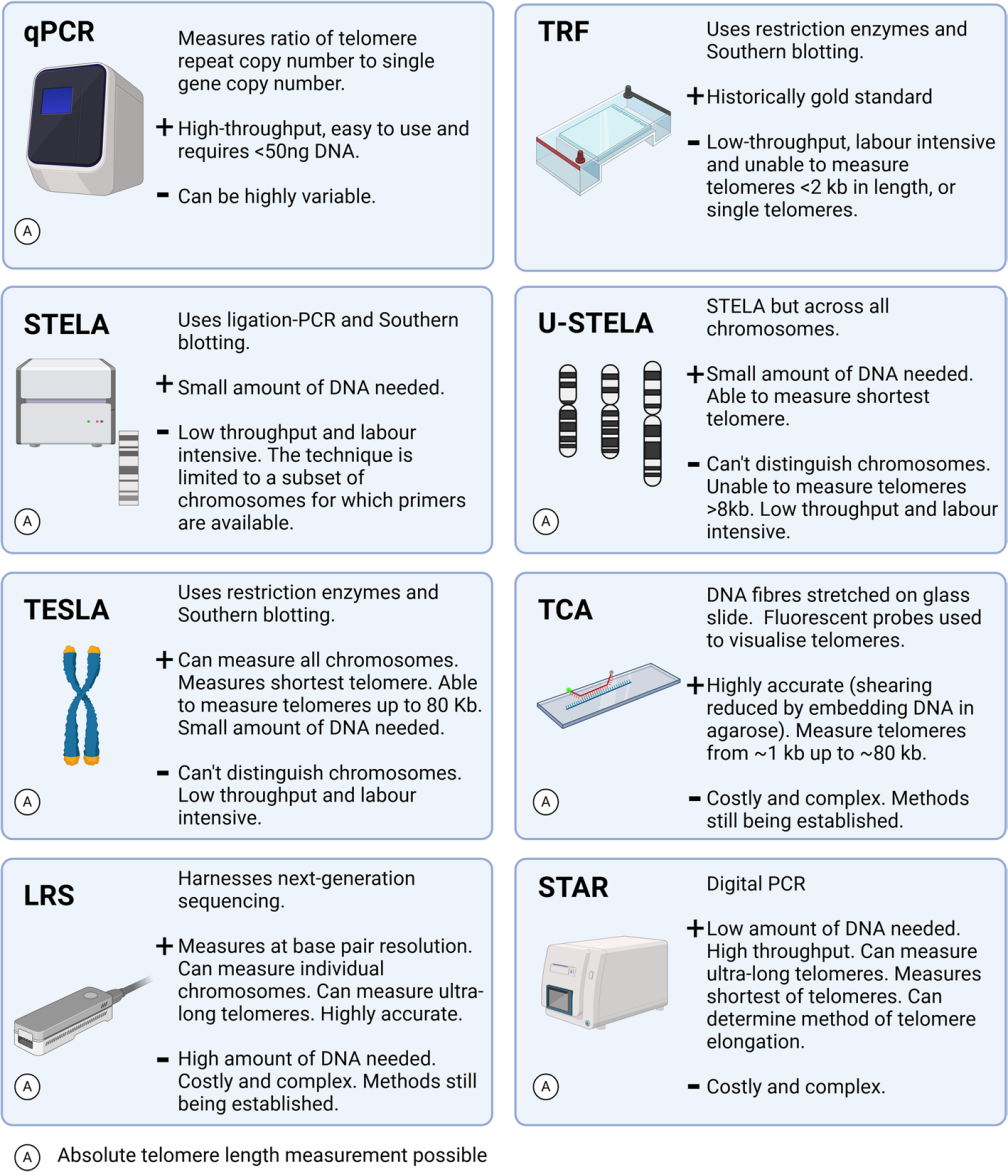
Summary of key telomere length measurement methods, including advantages (+) and drawbacks (-). Quantitative Polymerase Chain Reaction (qPCR), Terminal Restriction Fragment (TRF), Single TElomere Length Analysis (STELA), universal STELA (U-STELA), TElomere Shortest Length Assays (TELSA), Telomere Combing Assays (TCA), Long read sequencing (LRS), Single Telomere Absolute-length Rapid (STAR)

Telomere length (TL) has been investigated in non-cancerous, non-communicable diseases [2, 4, 5]. Many studies are correlative, lacking causality [6], perhaps due to generalised approaches historically employed to measure telomeres. A range of methodologies exist for measuring TL, with unique utilities and limitations [7] (Fig. 1). Some methods are higher throughput or cost-effective, others measure TL at single-base resolution or measure the shortest telomere. The shortest telomeres are important as they have been proposed as a key trigger for cellular senescence [8–10] and are associated with premature ageing or increased disease risk [11, 12]. Studies also differ with respect to tissues assessed; whole blood TL can be a useful proxy for TL in other tissues [12]. Researchers must choose appropriate methodology their study.

### Quantitative Polymerase Chain Reaction (qPCR)

qPCR is relatively simple, scalable, and requires small DNA quantities [13]. qPCR provides relative or absolute TL measurements [13, 14]. Monochrome Multiplex-qPCR (MM-qPCR) has advanced this method, reducing variability as measures from telomere (T) and single copy reference (S) primers come from the same well, increasing throughput, lowering cost, and improving accuracy [15]. Master mix composition may influence measurements [16], demonstrating standardisation is needed. Problems arise if the reference gene is duplicated, reduced, or removed, with suggestions qPCR should only be used in instances with chromosomal stability and regular karyotypes [6]. Despite advancements, variation is still an issue [7].

### Omega Quantitative Polymerase Chain Reaction (Ω-qPCR)

Ω-qPCR quantifies average TL at base-pair resolution [17], tagging telomeric DNA with “Ω -probes”. Hybridised and circularised Ω-probes are quantified via qPCR. An understanding of the ploidy and cell cycle status is required to obtain accurate results [17]. The utility in population-scale studies may provide opportunities for this fledgling approach.

### Terminal Restriction Fragment Analysis (TRF)

TRF is often considered the ‘gold-standard’ method for quantifying TL, first described in 1988 [18], detailed by Mender and Shay [19]. TRF involves degrading DNA so only telomeric repeats remain [19]. Remnants from the sub-telomeric region may remain, including TTAGGG repeats outside the telomeric region, which could disrupt measurements. It is important to decide a restriction enzyme cocktail that minimises sub-telomeric regions. Enzymes utilised vary between labs, making comparisons difficult and increasing variation [20]. The Telomere-to-Telomere human genome sequence [21], including sub-telomeric regions, should aid the selection of appropriate enzyme combinations. The development of commercially available kits has also aided standardisation. The necessity of large amounts of starting DNA (~ 3µg), labour intensity, average measurements, inability to detect short telomeres (< 2kb) and low-throughput has led to the technique being less-optimum for population-level studies, but useful as a control for testing novel techniques or in “proof-of-concept” studies.

### Single Telomere Absolute-length Rapid (STAR) Assay

STAR is a novel real-time high-throughput digital PCR technique for measuring absolute TL from as little as 1ng of DNA [22]. The technique measures individual telomeres from 0.2kb to 320kb in under 3 h. Restriction enzymes degrade non-telomeric DNA. Dilution and nanolitre compartments ensure that compartments generally contain one to zero telomere fragments. The utility of STAR may be best found within cancer studies, detecting whether cancer cells are using telomerase or the telomerase-independent “alternative lengthening of telomere” (ALT) pathway [22]. Approximately, 10–15% of tumours are ALT-positive; associated with poor prognoses [23]. STAR can detect four key markers of ALT: long telomeres, heterogeneity in TL, abundances of short telomeres, and the presence of extrachromosomal telomeric repeats (ECTR) [22], highlighting its potential biomarker utility. Whilst STAR was designed for the Fluidigm’s digital PCR chip, the use of other digital PCR systems with higher throughput (such as Qiagen’s QIAcuity system) [22] is yet to be explored.

### Single Telomere Length Analysis (STELA)

STELA allows telomere measurements on individual chromosomes using chromosome-specific, sub-telomeric primers, before applying Southern blotting [24]. STELA benefits from a low DNA requirements and shortest telomeres measurements [7]. It is highly labour intensive, relatively low-throughput, and limited to specific chromosomes [7]. Recent updates include high-throughput STELA (HT-STELA) [25] and universal STELA (U-STELA, measuring all chromosomes) [26]. U-STELA detects ultra-short telomeres [26, 27] but is poorly suited for telomeres above 8kb, and still suffers from low-throughput and high labour intensity [20].

### Telomere Shortest Length Assay (TeSLA)

TeSLA measures all telomeres, including the shortest, via TeSLA-T adaptor ligation (attached to the 3-prime G-rich telomere overhang), restriction enzyme digestion, TeSLA adaptor ligation (proximally tagging genomic and sub-telomeric sequences for primer annealing), PCR and Southern blotting [28]. TeSLA avoids detecting non-telomere telomeric repeats, has low starting material requirements (picograms of DNA [29]) and measures telomeres from below 1kb to 18kb [28]. TeSLA is burdened by low-throughput and high labour intensity [20]. It may be best suited for studies investigating changes in short telomeres over time, such as the accumulation of short telomeres with ageing [30].

### Telomere Combing Assay (TCA)

TCA measures the distribution and absolute length of telomeres [31], as well as dynamic TL changes. DNA samples are ‘combed’ onto glass slides with a constant stretch factor applied [31]. A telomere specific probe allows telomeres to be visualised via fluorescence microscopy [31]. Existing automated analysis tools lack robust reproducibility [32], reducing throughput. Advancements could increase applicability to population studies. TCA measure from ~ 1kb up to ~ 80kb. Accuracy reduction, found when analysing ultra-long telomeres, is partially mitigated through embedding samples in agarose to minimise DNA shearing [31]. TCA correlates well with other methods (qPCR (R^2^ = 0.93), Q-FISH (R^2^ = 0.91), TRF (R^2^ = 0.80)) [31]; however, literature demonstrating the technique is yet to be published. Q-FISH (quantitative fluorescence in situ hybridization) are well-established methods [7, 33, 34], allowing TL to be determined for individual chromosomes and single-cells [35]. Given the utility of FISH in a clinical setting (such as cancer diagnosis and prognosis) [36], this close correlation and TCAs ability to assess dynamic changes, suggests TCA may be suited to study TL variation alongside disease progression. TCA is highly sensitive and can measure subtle differences, including in telomere structure.

### Long-read telomere sequencing

The development of long-read sequencing technologies and bioinformatic pipelines has brought possibilities for measuring TL at each chromosome end, at single-base resolution. Oxford Nanopore (detecting differences in ionic current as bases pass through a pore [37]) revealed an absolute chromosome-specific telomere measurement pipeline, “Telo-Seq”, revealing intra-sample variation [38]. Telomere adaptors, complementary to the 3-prime overhang, are used to anneal sequencing primers which facilitate sequencing into the sub-telomeric region, alongside multiplexing [39]. Nanopore sequencing, via alternative protocols, has been used to measure telomeres in *S. cerevisiae* [40], mice [41] and humans [42].

Bionano is an alternative approach used to measure TL at individual chromosomes [43], using high-resolution florescent microscopy. This method is limited at specific chromosome regions (13p, 14p, 15p, 16p, 17p, 19q, 21p and 22q); however, utilising the Telomere-to-Telomere reference and future methodology changes may improve this.

PacBio sequencing has also achieved nucleotide resolution TL measurements using single-molecule real time (SMRT) sequencing, detecting the incorporation of fluorescently-labelled DNA bases [37, 44], functional in cultured cells and clinical samples. They highlighted heterogeneity in telomere variant sequences (TVSs) which may have implications in disease [44]. These TVSs are deviations from the canonical telomeric repeats ((TTAGGG)_n_). TVSs dispersed along telomeres can alter the binding of shelterin, a protein complex protecting chromosome ends. TVS distribution is unique to each individual and may be an inherited factor impacting telomere degradation, ageing and disease [44]. Telomere sequences (using ‘Telobait’ oligos) can be enriched prior to PacBio sequencing, with the option to multiplex [44].

A flexible TL analysis tool, “Telogator,” is useful for Oxford Nanopore and PacBio sequencing [45, 46], aligning reads to Telomere-to-Telomere reference genomes for accurate TL quantification, allele-specific TL and characterisation of telomere variant repeats (found between telomeres and sub-telomeres, composed of canonical repeats interspersed with blocks of TVSs) (https://github.com/zstephens/telogator2).

Long-read techniques are in their infancy, expensive and require high amounts of starting material. Studies have compared long-read sequencing methods [47, 48], with the ‘best’ method often use-case dependent [49]. Basecalling errors across Nanopore platforms and basecallers (not observed with PacBio) have been detected due to the repetitive nature of telomere repeats; however, a selective telomere-focused basecalling method has resolved such basecalling errors [37]. Oxford Nanopore has strengths in flexibility in scale and portability. For ultra-long telomeres, TCA or STAR may be more appropriate as even long-read sequencing reads (typically 20 to 30 kilobases in length) may not be long enough to span the entire telomere [46]. Combining sequencing methods can overcome biases and errors [50]. With the rate at which long-range sequencing is advancing [51], higher throughput coupled with better accuracy and refined pipelines, at a reduced cost, holds potential to transform the telomere field.

## Conclusion

To better understand the relationships between TLs, ageing, and disease, it is important that researchers choose the best approach. It is often difficult to draw comparisons between studies and identify robust outcomes from meta-analyses due to diverse methodologies; therefore, it is important to detail methods and develop standards. The Telomere Research Network (TRN) recently established international collaborative efforts to address these needs (https://trn.tulane.edu/).

Looking forward, understanding the role telomeres play in healthy ageing may reduce the future burden of our globally ageing population; however, findings must be robust and reproducible for translation into practice. There is no one-size-fits-all approach for measuring telomeres, but as techniques continue to improve and standardised pipelines become established, each methodological niche can address tailored research questions.

## Data Availability

No datasets were generated or analysed during the current study.

## Abbreviations

cDNA: Complementary DNA
COPD: Chronic obstructive pulmonary disease
ECTR: Extrachromosomal telomeric repeats
gDNA: Genomic DNA
HT-STELA: High-throughput STELA
kb: Kilobases
MM-qPCR: Monochrome Multiplex-qPCR
PDMS: Polydimethylsiloxane
Q-FISH: Quantitative Fluorescence In Situ Hybridization
qPCR: Quantitative Polymerase Chain Reaction
S: Single gene copy number
SMRT: Single-molecule real time
STAR: Single Telomere Absolute-length Rapid
STELA: Single TElomere Length Analysis
T: Telomere repeat copy number
TCA: Telomere Combing Assays
TeloTag: Biotinylated oligonucleotide
TESLA: TElomere Shortest Length Assays
TL: Telomere length
TRF: Terminal Restriction Fragment analysis
TVSs: Telomeric variant sequences
U-STELA: Universal STELA
Ω-qPCR: Omega qPCR
